# Motility is required for the competitive fitness of entomopathogenic *Photorhabdus luminescens *during insect infection

**DOI:** 10.1186/1471-2180-8-168

**Published:** 2008-10-03

**Authors:** Catherine A Easom, David J Clarke

**Affiliations:** 1 Department of Microbiology, University College Cork, Ireland; 2 Department of Biology and Biochemistry, University of Bath, BA2 7AY, UK

## Abstract

**Background:**

*Photorhabdus *are motile members of the family Enterobactericeae that are pathogenic to insect larvae whilst also maintaining a mutualistic interaction with entomophagous nematodes of the family Heterorhabditiae. The interactions between *Photorhabdus *and its hosts are thought to be an obligate part of the bacteria's life-cycle in the environment. Motility often plays a key role in mediating bacteria-host interactions and, in this study, we were interested in characterising the role of motility in the *Photorhabdus*-nematode-insect tripartite association.

**Results:**

We constructed deletion mutants of *flgG *(blocking flagella production) and *motAB *(blocking flagella rotation) in *P. luminescens *TT01. Using these mutants we show that both the Δ*flgG *and Δ*motAB *mutants are equally as good as the wild-type (WT) bacteria in killing insects and supporting nematode growth and development suggesting that flagella production and motility are not required for pathogenicity or mutualism. However we show that the production of flagella is associated with a significant metabolic cost during growth on agar plates suggesting that, although not required for pathogenicity or mutualism, there must be a strong selective pressure to retain flagella production (and motility) during the interactions between *Photorhabdus *and its different hosts. To this end we show that both the Δ*flgG *and Δ*motAB *mutants are out-competed by WT *Photorhabdus *during prolonged incubation in the insect revealing that motile bacteria do have a fitness advantage during colonisation of the insect larva.

**Conclusion:**

This is the first report of a role for motility in *Photorhabdus *and we show that, although not required for either pathogenicity or mutualism, motility does contribute to the competitive fitness of *Photorhabdus *during infection of the insect (and, to a lesser extent, the nematode). This adaptive function is similar to the role ascribed to motility in mammalian pathogens such as uropathogenic *Escherichia coli *(UPEC). Therefore, in addition to describing a role for motility in *Photorhabdus*, this study reinforces the relevance and utility of this bacterium as a model for studying bacteria-host interactions.

## Background

*Photorhabdus *are Gram negative bacteria that are highly virulent pathogens of a wide variety of insect larvae whilst also maintaining a mutualistic interaction with nematodes from the family *Heterorhabditidieae*. *Photorhabdus *normally colonise the intestinal tract of the infective stage of the nematode, the infective juvenile (IJ). The IJs are a non-feeding stage of the nematode that lives in the soil where they actively seek out potential larval hosts. On finding a suitable host the IJ enters the larva and the bacteria are regurgitated into the insect hemolymph. *Photorhabdus *actively circumvent the insect innate immune system by inhibiting and adapting to the humoral response whilst also suppressing phagocytosis by circulating haemocytes [1-3]. During infection of the insect the bacteria grow exponentially, producing a wide range of toxins and hydrolytic exoenzymes that are responsible for the death and subsequent bioconversion of the insect larva into a nutrient soup that is ideal for nematode growth and development [4,5]. The nematodes feed on the bacterial biomass within the insect cadaver and develop through juvenile (J1–J4) stages to form adult males and females. After several rounds of reproduction the J1 stage nematodes receive uncharacterised environmental cues that stimulate the development of IJs. At this point the developing IJs are colonised by *Photorhabdus *before they emerge from the insect cadaver to find new hosts (for recent reviews see [6,7]).

Many bacteria are motile through the action of large complex protein assemblages called flagella. The production and function of flagella are best studied in the enteric bacteria *Escherichia coli *and *Salmonella enterica *serovar Typhimurium where it has been shown that the expression of genes required for flagella-mediated motility (and chemotaxis) is controlled by a complex regulatory network [8,9]. Flagellum-mediated motility often plays a key role in mediating different bacteria-host interactions. For example motility is important during the colonisation of the squid by *Vibrio fischeri *and also during the infection of mammals by both *Salmonella *and *E. coli *[10-14]. *Photorhabdus *are motile through the action of peritrichously arranged flagella and we hypothesised that motility must play some role in the interactions between *Photorhabdus *and its invertebrate hosts. This was based on the principle that unused or redundant traits in bacteria will be lost over time [15]. Therefore we constructed specific flagellum-minus (Fla-) and non-motile (Mot-) mutants of *Photorhabdus *and, using these mutants, we show that motility is not required for either pathogenicity or mutualism. However we do show that WT bacteria out-compete non-motile mutants during prolonged incubation in insect cadavers suggesting that motility confers a fitness advantage during colonisation of the insect.

## Results

### Construction of mutations in *flgG *and *motAB*

**Figure 1:**
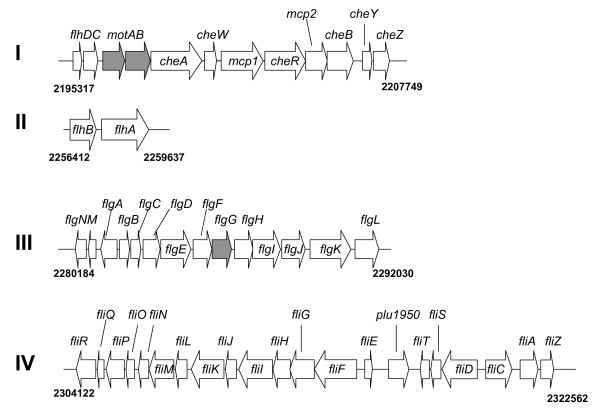
**The genetic loci encoding the genes required for flagella production and motility in *P. luminescens *TT01**. By comparison with the *E. coli *and *Salmonella *genomes (available at ColiBase ) we identified 4 genetic loci (labelled I-IV) predicted to be involved in the production of flagella and chemotaxis in *Photorhabdus*. The numbers shown at beginning and end of each locus represents the genetic location on the *Photorhabdus *genome (according to PhotoList ). The genes deleted in this study (*motAB *and *flgG*) are indicated in grey.

**Figure 2:**
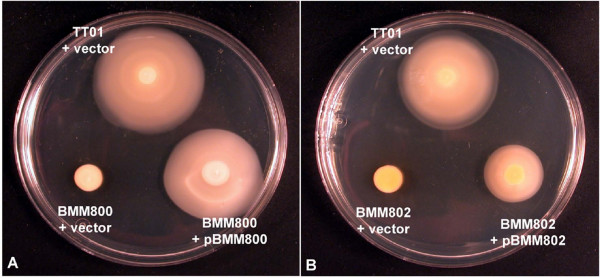
**Swimming motility of *P. luminescens***. The WT was transformed using the plasmid pTRC99a (TT01 + vector) and the mutants BMM800 (Δ*flgG*) and BMM802 (Δ*motAB*) were transformed using pTRC99a (BMM800 + vector and BMM802 + vector) or either pBMM800 (BMM800 + pBMM800) or pBMM802 (BMM802 + pBMM802). Cells were grown overnight at 30°C in LB broth (+ Amp) and diluted to an OD_600 _= 1.0 in fresh LB before 5 μl was spotted onto the surface of a swim agar plate. Plates were incubated at 30°C for 44 h before motility was scored.

The genome sequence of *P. luminescens *TT01 is available and, by comparison with the closely related genome of *E. coli*, the TT01 genome is predicted to contain 49 genes required for the production and assembly of functional flagella and chemotaxis [16]. The motility-associated genes are found as 4 distinct genetic loci on a large (approx. 130 Kb) fragment of TT01 genomic DNA stretching from nucleotide 2195317 to 2322562 (Fig. 1). To examine the role of motility in *P. luminescens *TT01 we constructed mutations in genes that are known to be required for motility in other bacteria, *flgG *and *motAB*. The *flgG *gene encodes the distal rod protein of the flagellar hook-basal body (HBB) and mutations in *flgG *would be expected to prevent completion of this structure and, therefore, flagella assembly (Fla-). The *motAB *genes encode the motor-force generator that is required for rotation of the flagella and strains carrying mutations in *motAB *can assemble normal flagella but the flagella cannot rotate (Mot-). Using a strategy that results in the construction of unmarked, non-polar deletions we completely removed the *flgG *gene such that only the start and stop codons remained. However the *motAB *operon overlaps with the downstream *cheA *gene and, to prevent any polar affects on *cheA *expression, the last 20 nucleotides of the *motB *gene (containing the predicted ribosome-binding site for the *cheA *gene) were not deleted. We confirmed that BMM800 (Δ*flgG*) and BMM802 (Δ*motAB*) were non-motile using swim agar and that motility could be restored by the in trans expression of the respective genes from a plasmid i.e. pBMM800 (*flgG*) and pBMM802 (*motAB*) respectively (Fig. 2).

### Motility is required for attachment to surfaces

**Figure 3:**
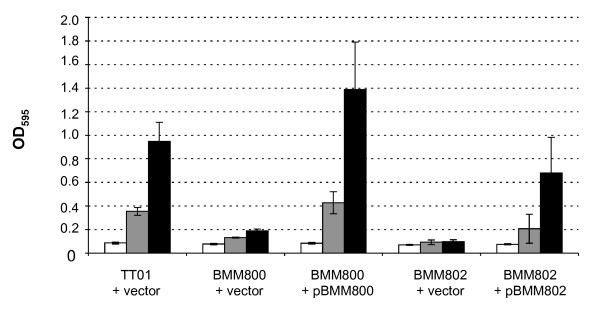
**Biofilm formation by *P. luminescens***. The cells (as indicated; see legend for Figure 2) were grown overnight at 30°C in LB broth (+ Amp). The OD_600 _of the culture was adjusted to 0.05 and 200 μl was added to the well of a 96-well Costar^® ^PP microtitre plate. The plates were incubated for the appropriate time at 30°C before staining with crystal violet to quantify bacterial attachment. The results shown are the mean ± SD of 3 experiments.

In *E. coli *(and other bacteria) it has been shown that flagella are important for bacterial attachment to abiotic surfaces [17-20]. Therefore the wells of a polypropylene microtitre plate were inoculated with TT01, BMM800 (Δ*flgG*) and BMM802 (Δ*motAB*) and the plate was incubated at 30°C without shaking for 24 h, 48 h and 72 h. Bacterial attachment was quantified using crystal violet and it is clear that both the Δ*flgG *and Δ*motAB *mutants were severely affected (5–10-fold) in their ability to attach to the walls of the microtitre plates when compared to the WT bacteria (Fig. 3). Attachment was restored to the mutants carrying a plasmid expressing the appropriate gene(s). Therefore, as in other enteric bacteria, motility is required for attachment of *Photorhabdus *to abiotic surfaces.

### There is a cost associated with the production of flagella

**Figure 4:**
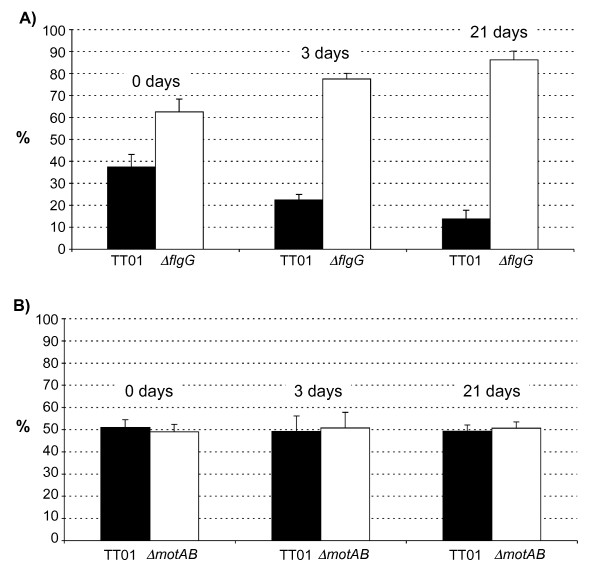
**Competition assays on agar plates**. Equal numbers of A) TT01 and BMM800 (Δ*flgG*) and B) TT01 and BMM802 (Δ*motAB*) were mixed and inoculated onto each of 5 lipid agar plates. The proportion (%) of motile:non-motile bacteria was determined in the initial mixture before inoculation and this is reported as Day 0. At the other time points the proportion of motile:non-motile bacteria was determined on each of the 5 individual plates. The results shown are the mean ± SD of 3 experiments.

Although flagella are generally not required for growth their production and assembly can be costly to the cell in terms of the utilisation of resources [21]. To determine whether the production of flagella is costly to *Photorhabdus *we set up a competition experiment whereby lipid agar plates were inoculated with 50:50 mixtures of WT bacteria with either the Δ*flgG *or the Δ*motAB *mutant. The plates were incubated at 30°C and the relative abundance of each bacterial strain on the agar plate was measured at time intervals post-inoculation i.e. 3 days and 21 days. In preliminary tests using LB broth we established that the growth rates of the Δ*flgG *and the Δ*motAB *mutants were identical to WT (data not shown). However, when grown for an extended period of time on lipid agar plates, we observed that the Δ*flgG *mutant was present at higher levels than the WT (a ratio of 85:15 on day 21) on the plate (Fig. 4A). On the other hand the Δ*motAB *mutant maintained a 50:50 ratio with the WT throughout the 21 days (Fig. 4B). Therefore there is a measurable cost associated with the production of flagella, but not motility per se, during *Photorhabdus *growth in vitro.

### Motility is required for the competitive fitness of *Photorhabdus *during insect colonization

**Figure 5:**
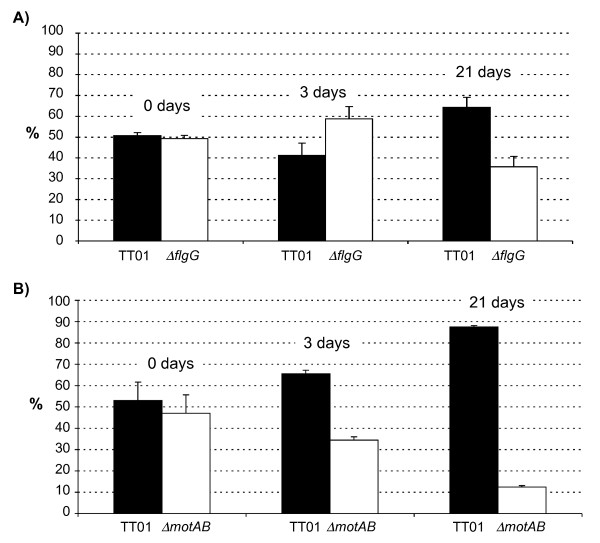
**Competition assays in the insect**. Equal numbers of A) TT01 and BMM800 (Δ*flgG*) and B) TT01 and BMM802 (Δ*motAB*) were mixed and injected into *G. mellonella *larvae. The proportion (%) of motile:non-motile bacteria was determined in the initial mixture before injection and this is reported as Day 0. At Day 3 and Day 21 the proportion of motile:non-motile bacteria was determined in each of 10 individual insects. The results shown are the mean ± SD of 3 experiments.

To test for a possible role during insect virulence, we injected 200 cfu of TT01, BMM800 (Δ*flgG*) or BMM802 (Δ*motAB*) into final instar *Galleria mellonella *larvae. The LT_50 _(i.e. time taken to kill 50% of the insect larvae) values of the WT and mutant bacteria were similar showing that motility is not required for *Photorhabdus *virulence against insects (data not shown). To test whether motility might confer an advantage at some point during infection of the insect we injected a 50:50 mixture of TT01 with either the Δ*flgG *or the Δ*motAB *mutant into *G. mellonella *larvae and incubated the larvae at 25°C for either 3 or 21 days (all insects were dead after 2 days). The insects were then homogenised and the proportion of motile to non-motile bacteria was determined using swim agar, as described. After 3 days in the insect the Δ*flgG *mutant was present at slightly higher levels than the WT but this trend was reversed after 21 days at which time the WT predominated in the insect cadaver (Fig. 5A). Similarly the WT had almost completely out-competed the Δ*motAB *mutant after 21 days in the insect (Fig. 5B). Therefore motility confers a fitness advantage to *Photorhabdus *during the prolonged incubation in the insect normally experienced by these bacteria as part of their life-cycle. This would suggest that the cost associated with the production of flagella is offset by the ability of *Photorhabdus *to be motile in the insect.

### Motility affects the ability of *Photorhabdus *to colonise the nematode

**Figure 6:**
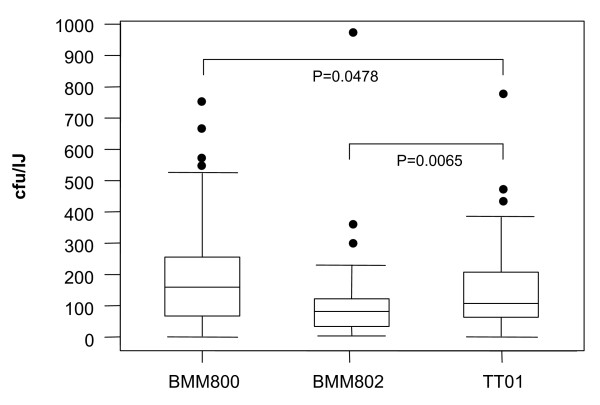
**Bacterial levels in IJ stage nematodes**. IJ nematodes were harvested from lipid agar plates inoculated with TT01, BMM800 (Δ*flgG*) or BMM802 (Δ*motAB*). Individual IJs were crushed and the homogenate was plated on LB (Rif) plates to determine the bacterial population per IJ (cfu/IJ). Three independent overnight cultures for each strain were prepared and each overnight was inoculated onto 4 lipid agar plates. After 21 days incubation at 25°C 10 IJs from each plate (n = 120 for each bacterial strain) were homogenised and the number of cfu/IJ was determined by plating the homogenate on LB agar (Rif). Any nematodes that were not colonised were not included in further analyses. Therefore, for IJs grown on WT bacteria n = 100, for IJs grown on the Δ*flgG *mutant n = 102 and for IJs grown on the Δ*motAB *mutant n = 98. The data from these nematodes was analysed and compared to WT using the non-parametric Mann-Whitney U test and the appropriate P values are shown. The data is presented using a Box and Whisker format that shows the median value (horizontal line), the upper and lower quartiles (boxed) and the range of the data (whiskers). The remaining "outliers" are shown as solid circles.

To test for a role for motility during the interaction with the nematode we inoculated lipid agar plates with overnight cultures of TT01, BMM800 (Δ*flgG*) or BMM802 (Δ*motAB*). After 3 days at 25°C the bacterial biomass on the plates was seeded with 50 surface-sterilised IJ nematodes and incubated, in the dark, at 25°C for a total of 21 days. During this time 3 aspects of the bacteria-nematode interaction are routinely monitored: 1) the fraction of inoculated IJs that exit diapause to develop into adult hermaphrodites (i.e. this is called IJ recovery and it is an indicator of the ability of the bacteria to produce the signals required to stimulate IJ recovery); 2) the total number of new generation IJs that are recovered after 21 days (i.e. this is called the IJ yield and it is an indicator of the ability of the bacteria to support nematode growth and reproduction) and 3) IJ colonisation by the bacteria (required for the continuation of the mutualism between bacteria and nematode). We did not observe any defect in either IJ recovery or IJ yield suggesting that bacterial motility does not make a significant contribution to nematode growth and development (data not shown). On the other hand we did observe that the Δ*flgG *mutant was present in the IJ at levels significantly higher than the WT (median for Δ*flgG *= 159 cfu/IJ compared to median for WT = 105 cfu/IJ; *P *= 0.0478) whilst, in contrast, the Δ*motAB *mutant was present in the IJ at significantly lower levels than the WT (median for Δ*motAB *= 82 cfu/IJ compared to median for WT = 105 cfu/IJ; *P *= 0.0065) (Fig. 6). Therefore, although not required for colonisation, the ability to produce flagella and motility do appear to have contrasting affects on the final number of bacteria present in the IJ.

## Discussion

*Photorhabdus *are highly virulent to a wide range of insect larvae and, following insect death the bacteria must remain in the insect cadaver at high densities for extended periods of time (up to 2–3 weeks under optimal conditions) to facilitate nematode growth and development. We have shown that, although neither flagella production nor motility is required for pathogenicity, there is a significant advantage to being motile during the normally prolonged incubation in the insect.

Our data show that, on agar plates, the Δ*motAB *mutant is as competitive as the WT suggesting that there is no perceived benefit to being motile in this environment. This was not unexpected as the concentration of agar used would prevent cells from swimming or swarming, whether they are motile or not, thus rendering the Δ*motAB *mutant neutral in this environment. On the other hand, the Δ*flgG *mutant has a considerable advantage over WT suggesting that there is a metabolic cost associated with the production of flagella. In a recent study Fontaine *et al *(2008) showed that the reduced mortality associated with a *fliA *mutation in *E. coli *was probably due to decreased internal cell stress due to the absence of physical destabilisation of the membrane [21]. Therefore the cost associated with flagella production in *Photorhabdus *may be due to the utilisation of resources and energy for the production and/or function of the flagella or it may be due to the stresses associated with assembly of the flagella through the cellular membrane. In the insect this trend is reversed and the Δ*flgG *cells are now disadvantaged during extended growth (i.e. 21 days). This would suggest that flagella production benefits the bacteria during insect infection. Therefore the costs of flagella production (and presumably motility) appear to be offset during extended incubation in the insect. In support of this, the Δ*motAB *mutant, that still bears the cost of producing flagella and yet does not derive any benefit associated with motility in the insect, is present at much lower levels than the Δ*flgG *mutant in competition assays after 21 days.

The frequency of motile cells observed throughout the growth of *Photorhabdus *under normal culturing conditions (i.e. shaking at 30°C in LB broth) is very low (<< 1%) (our unpublished data). During growth under static conditions the frequency of motility in the population increased reaching a maximum of 30% at 16 h post-inoculation before rapidly decreasing (data not shown). Therefore motility is restricted, both spatially and temporally, during the growth of *Photorhabdus*. This limited exposure of motility to any selection pressure in the environment could explain the relatively modest differences in fitness observed in this study.

In uropathogenic *E. coli *(UPEC) motility has been shown to contribute to the fitness of the bacteria during colonisation of the urinary tract [22]. Indeed recent studies have shown that motility facilitates the movement of UPEC to the upper urinary tract [12]. Moreover motility has been shown to be required for *Salmonella *to access high-energy nutrients found at sites of inflammation in the mammalian gut [13]. In the same way we expect that motility in *Photorhabdus*, a closely related bacterium, will facilitate the movement of the bacteria throughout the insect cadaver thus enabling invasion of nutrient-rich niches that facilitate growth of the motile strain. In this regard *Photorhabdus *would also be expected to undergo chemotaxis and the genome is predicted to encode a complete Che signalling system in addition to 2 methyl-accepting chemotaxis proteins i.e. MCPs (see Fig. 1). However the role of chemotaxis was not investigated in this study.

We have shown that neither flagella production nor motility is required for the mutualistic association between *Photorhabdus *and the *Heterorhabditis *nematode. *Photorhabdus *has 2 roles during the interaction with the nematode: 1) the bacteria must provide nutrients for the nematode and 2) the bacteria must colonise the IJ. The nematodes feed on the *Photorhabdus *biomass that is present either in the insect or on agar plates and, therefore, the nematodes obtain a substantial part of their required nutrients directly from the bacteria. We did not see any differences in the growth and development of the nematodes growing on agar plates with the WT bacteria compared to the Δ*flgG *and Δ*motAB *mutants. Indeed infecting insects with IJs colonised with either Δ*flgG *or Δ*motAB *mutants resulted in an IJ yield (a quantitative indicator of the ability of the bacteria to support nematode growth and development) very similar to that observed with IJs colonised with WT bacteria (data not shown). Therefore flagella production and motility do not appear to play any role in the nutritional interaction between *Photorhabdus *and the nematodes either on agar plates or in the insect.

*Photorhabdus *are maternally transmitted from the hermaphrodite stage nematode (i.e. the mother) to the developing IJ [23]. As the *Heterorhabditis *nematodes feed on *Photorhabdus *some viable bacteria enter the lumen of the gut of the hermaphrodite and attach to specific cells in the distal region of the gut (specifically the INT9 cells). The bacteria infect the neighbouring rectal glands cells and replicate within vacuoles. The rectal gland cells rupture, releasing *Photorhabdus *into the body cavity of the hermaphrodite where the bacteria encounter and colonise the developing IJs [23]. The IJ is initially colonised by 1–2 bacteria that subsequently replicate, resulting in a final population of approximately 100 cfu of WT bacteria per IJ. The proportion of IJs colonised by the Δ*flgG *and Δ*motAB *mutants is the same as the WT (i.e. approx. 80% in all cases) suggesting that attachment to the IJ, and presumably infection of the hermaphrodite, is independent of flagella and/or motility. On the other hand, the final population level of *Photorhabdus *within the IJ is significantly altered in nematodes that are cultured on either the Δ*flgG *or the Δ*motAB *mutant strain. Therefore IJs that have been grown on the Δ*flgG *mutant carry, on average, a bacterial population that is 50% greater than the population within IJs cultured on WT bacteria. In contrast the Δ*motAB *mutant does not reach population levels within the IJ that are equivalent to the WT suggesting that the production of non-functional flagella negatively influences growth in the nematode. These results might be explained in terms of the metabolic cost associated with the production of flagella. Therefore, in the absence of flagella production, the Δ*flgG *mutant may be able to put more of the limited resources available within the nematode into biomass production and division. On the other hand the Δ*motAB *mutant still bears the cost of producing flagella although these are non-functional. Interestingly the fact that the Δ*motAB *mutation is not neutral, in terms of IJ colonisation, suggests that *Photorhabdus *are likely to be motile at some point during the colonisation of the nematode. The bacteria initially colonise the proximal end of the IJ gut and one possibility is that motility may facilitate exploration of the distal regions of the gut thus allowing the bacteria to make better use of the limited resources available within the nematode.

## Conclusion

In this study we show that there is a significant metabolic cost associated with the production of flagella (and motility) in *Photorhabdus*. Nonetheless *Photorhabdus *are motile suggesting that motility is an adaptive trait that is under powerful positive selection in the environments where *Photorhabdus *is normally found i.e. in the insect and nematode. In this study we show that, although motility is not required for either pathogenicity or mutualism, this trait is advantageous during the interactions between *Photothabdus *and both of its invertebrate hosts. Therefore, in addition to describing a role for motility in *Photorhabdus*, this work also highlights the functional overlap between pathogenicity and mutualism and reinforces the utility of *Photorhabdus *as a model for studying these different bacteria-host interactions.

## Methods

### Bacterial strains and culture conditions

A spontaneous rifampicin-resistant mutant of *Photorhabdus luminescens *subsp. *luminescens *was used as the wild-type (WT) in all experiments [2]. The bacteria were cultured in LB broth or on LB agar (LB broth plus 1.5% (w/v) agar) at 30°C for *P. luminescens*. Unless otherwise stated all LB agar plates were supplemented with 0.1% (w/v) pyruvate [24]. *Escherichia coli *S17-1 (λpir) and *E. coli *EC100 (Epicentre) were cultured at either 30°C or 37°C as indicated. Swim agar is LB broth plus 0.3% (w/v) added agar. When required antibiotics were added at the following concentrations: ampicillin (Ap), 100 μg ml^-1^; chloramphenicol (Cm), 20 μg ml^-1 ^and rifampicin (Rif), 50 μg ml^-1^.

### Construction of deletion mutants

The *flgG *and *motAB *genes were deleted using a previously described procedure [25]. This method results in the construction of unmarked and non-polar deletion mutation of the selected gene(s). For the deletion of each gene fragment A (approx. 600 bp upstream of the target gene) and fragment B (approx. 600 bp downstream of the target gene) were amplified by PCR using KOD Hi-Fi polymerase (Novagen) and the primer pairs KO1 + KO3 (for fragment A) and KO2 + KO4 (for fragment B). The primer sequences are listed in Table 1. The fragments were purified using the Qiagen PCR clean up kit, combined and subjected to 10 cycles of primerless PCR to allow the polymerase to anneal fragments A and B (as KO3 and KO4 are complimentary). The annealed fragment was used as a template in a final PCR, using the appropriate KO1 and KO2 primers, to enrich for a full-length fragment. The different KO1 and KO2 primers incorporate restriction sites to facilitate cloning into the suicide vector pDS132 [26]. The resulting pDS132-flgG and pDS132-motAB plasmids were isolated and used to transform *E. coli *S17-1 λpir (the donor) for conjugation into *P. luminescens *TT01 (the recipient). Conjugation and selection of the appropriate exconjugants was carried out as described previously [25]. Deletion mutants were then identified by subjecting selected exconjugants to colony PCR using the KO5 and KO6 primers (a deletion will result in a PCR product of a predictable size). The amplification product of each mutant was sequenced to confirm the integrity of the knock out allele.

**Table 1:** Primers used for the construction of the Δ*flgG *and Δ*motAB *mutants

Primer	Sequence (5' – 3') ^a^
KO1flgG	ATTATGCATGCCGATGAACTATACCTCTCGTCCG
KO2flgG	ATATAGAGCTCGCCAGTAACCGATCGACTGTCACG
KO3flgG	GACGATTAGGTTACATCGGTTTTATCCTCTGTGTC
KO4flgG	GGATAAAACCGATGTAACCTAATCGTCAATCAG
KO5flgG	GCAACTGGCTGCTTTGCGAGC
KO6flgG	GTCCGTGGGTTAACGACACCG
KO1motAB	TATGCATGCGTTACAAATGCTTGAAAGTGAAACAC
KO2motAB	ATATAGAGCTCGTGGCTGATGTTACCAATGATGC
KO3motAB	CGTTACTTTGTCACCTTGGTCGCACGCGATATCCTTTAGC
KO4motAB	GCTAAAGGATATCGCGTGCGACCAAGGTGACAAAGTAACG
KO5motAB	GCAGCTTACTAGGGAATCTCGAGTGG
KO6motAB	CTGCCCGTTGACGTGGCGATCCCG

^a ^restriction sites are underlined

### Cloning of the *flgG *and *motAB *genes

The *flgG *and *motAB *genes were amplified from *P. luminescens *TT01 genomic DNA by PCR using KOD polymerase. The primers used for *flgG *were CAT001 (5'-TAAAACCCATGGTCCGATCATTATGGATTGC-3') and CAT002 (5'-GCTGGATCTAGATTATAACTGAGTCAGTTTTTGTAGC-3') and for *motAB *CAT003 (5'-GATATCCCATGGTAGTACTTTTAGGATATATC-3') and CAT004 (5'-GCAGTGTCTAGATTACTTTGTCACCTTGGTCGG-3'). The *flgG *and *motAB *PCR fragments were digested with *Nco*I and *Xba*I and cloned into pTRC99a (Amersham Pharmacia Biotech) resulting in pBMM800 and pBMM802, respectively. The integrity and accuracy of all plasmid clones was verified by DNA sequencing.

### Biofilm formation

The capacity of *P. luminescens *to form biofilms was assessed by measuring bacterial attachment to a plastic surface [2]. Strains were grown overnight in LB broth, diluted to OD_600_= 0.05 in fresh LB and 200 μl of the cell suspension was aliquoted in triplicate, into the wells of a Costar^® ^polypropylene (PP) 96-well microtitre plate. The plates were sealed with a gas permeable membrane and incubated, without shaking in a saturated environment to prevent evaporation, at 30°C. At the appropriate time the planktonic cells were removed by aspiration and the wells were washed with 1× phosphate buffered saline (PBS). To observe biofilm formation 250 μl of 0.1% (w/v) crystal violet (CV) was added to each well and the plates were incubated at room temperature for 20 min before rinsing 3 times with 1 × PBS. To quantify biofilm formation the CV was dissolved in 250 μl of 95% ethanol and the CV concentration was determined by measuring the OD_595 _using a Genios (Tecan) plate reader.

### Pathogenicity assays

The pathogenicity of *P. luminescens *was assessed using *Galleria mellonella *larvae (the Greater Wax Moth) as the model insect host. The *G. mellonella *larvae were purchased from Livefood (UK). Briefly overnight cultures of *P. luminescens *TT01 were washed 3 times in 1 × PBS before the OD_600 _was adjusted to 1.0 as this has been shown to be equivalent to 4 × 10^8 ^cfu ml^-1 ^(our unpublished data). The culture was diluted with 1 × PBS to give cell density of 2 × 10^4 ^cfu ml^-1 ^and 10 μl (equivalent to 200 cfu) was injected into the hemolymph of a *G. mellonella *larva using a Hamilton syringe and a BD Microlance™ 3 30G × 1/2" needle. For competition assays the WT and mutant strain were grown overnight in LB at 30°C and equal numbers of cells from each culture were mixed and subsequently diluted so that 200 cfu could be injected into each insect larva. The proportion of WT and mutant bacteria in the injection mixture was assessed by plating an aliquot of the mixture onto LB (Rif) agar followed by patching 100 colonies onto swim agar. To determine the proportion of WT and mutant bacteria in the insect an infected insect larvae was surface sterilised by dipping in ethanol and passing the insect through a Bunsen flame before quickly plunging it into 5 ml sterile 1 × PBS in a universal tube. The insect cadaver was sliced open using a sterile scalpel blade and homogenised by adding 7 (3–4 mm) sterile glass beads to the tube, followed by vortexing for 2 min. The supernatant, containing the bacteria, was plated onto LB (Rif) agar and the proportion of motile/non-motile bacteria was determined by patching 50 colonies onto swim agar.

### *In vitro *symbiosis assays

An aliquot of 50 μl of an overnight culture diluted to an OD_600 _= 1.0 of the appropriate bacteria was spread, in a Z pattern, onto the surface of a lipid agar plate using an inoculating loop. The plates were incubated at 30°C for 3 days before adding 50 surface sterilised IJ nematodes to the bacterial biomass. Nematodes were surface-sterilised by washing in a solution (0.4% (w/v)) of hyamine (Sigma). Nematode recovery was assessed 7 days after addition of IJs by counting the number of hermaphrodites on the lipid agar plate. The new generation of IJs migrate to the lid of the Petri dish and, after 21 days, these nematodes were collected, by washing the lid with PBS to a final volume of 50 ml, and the number of IJs present (i.e the IJ yield) was determined. In competition assays the assays were the same except that the lipid agar plates were inoculated with equal numbers of the WT and mutant bacteria. At 3 and 21 days post-inoculation the bacterial biomass was scraped off the plate and the proportion of motile/non-motile bacteria was determined (as before). Colonisation levels in the IJ were determined by crushing single, surface-sterilised IJ nematodes in 100 μl 1 × PBS using a hand-held homogeniser and plating the homogenate onto LB (Rif) agar.

## Authors' contributions

CAE undertook all of the experiments described in this manuscript. DJC conceived of the study, designed the experiments and drafted the manuscript. All authors read and approved the final manuscript.

## References

[B1] Brugirard-Ricaud K, Duchaud E, Givaudan A, Girard PA, Kunst F, Boemare N, Brehelin M, Zumbihl R (2005). Site-specific antiphagocytic function of the *Photorhabdus luminescens *type III secretion system during insect colonization. Cell Microbiol.

[B2] Bennett HPJ, Clarke DJ (2005). The *pbgPE *operon in *Photorhabdus luminescens *is required for pathogenicity and symbiosis. J Bacteriol.

[B3] Kim Y, Ji D, Cho S, Park Y (2005). Two groups of entomopathogenic bacteria, *Photorhabdus *and *Xenorhabdus*, share an inhibitory action against phospholipase A2 to induce host immunodepression. J Invertebr Pathol.

[B4] Waterfield NR, Bowen DJ, Fetherston JD, Perry RD, ffrench-Constant RH (2001). The tc genes of *Photorhabdus*: a growing family. Trends Microbiol.

[B5] Daborn PJ, Waterfield N, Blight MA, Ffrench-Constant RH (2001). Measuring virulence factor expression by the pathogenic bacterium *Photorhabdus luminescens *in culture and during insect infection. J Bacteriol.

[B6] ffrench-Constant R, Waterfield N, Daborn P, Joyce S, Bennett H, Au C, Dowling A, Boundy S, Reynolds S, Clarke D (2003). *Photorhabdus*: towards a functional genomic analysis of a symbiont and pathogen. FEMS Microbiol Rev.

[B7] Goodrich-Blair H, Clarke DJ (2007). Mutualism and pathogenesis in *Xenorhabdus *and *Photorhabdus*: two roads to the same destination. Mol Microbiol.

[B8] Aldridge P, Hughes KT (2002). Regulation of flagellar assembly. Curr Opin Microbiol.

[B9] Chevance FF, Hughes KT (2008). Coordinating assembly of a bacterial macromolecular machine. Nat Rev Microbiol.

[B10] Millikan DS, Ruby EG (2002). Alterations in *Vibrio fischeri *motility correlate with a delay in symbiosis initiation and are associated with additional symbiotic colonization defects. Appl Environ Microbiol.

[B11] Gauger EJ, Leatham MP, Mercado-Lubo R, Laux DC, Conway T, Cohen PS (2007). Role of motility and the *flhDC *operon in *Escherichia coli *MG1655 colonization of the mouse intestine. Infect Immun.

[B12] Lane MC, Alteri CJ, Smith SN, Mobley HLT (2007). Expression of flagella is coincident with uropathogenic *Escherichia coli *ascension to the upper urinary tract. Proc Natl Acad Sci (USA).

[B13] Stecher B, Barthel M, Schlumberger MC, Haberli L, Rabsch W, Kremer M, Hardt W-D (2008). Motility allows *S. *Typhimurium to benefit from the mucosal defence. Cell Microbiol.

[B14] Millikan DS, Ruby EG (2004). *Vibrio fischeri *flagellin A is essential for normal motility and for symbiotic competence during initial squid light organ colonization. J Bacteriol.

[B15] Hall AR, Colegrave N (2008). Decay of unused characters by selection and drift. J Evol Biol.

[B16] Duchaud E, Rusniok C, Frangeul L, Buchrieser C, Givaudan A, Taourit S, Bocs S, Boursaux-Eude C, Chandler M, Charles JF (2003). The genome sequence of the entomopathogenic bacterium *Photorhabdus luminescens*. Nat Biotechnol.

[B17] O'Toole GA, Kolter R (1998). Flagellar and twitching motility are necessary for *Pseudomonas aeruginosa *biofilm development. Mol Microbiol.

[B18] Pratt LA, Kolter R (1998). Genetic analysis of *Escherichia coli *biofilm formation: roles of flagella, motility, chemotaxis and type I pili. Mol Microbiol.

[B19] Harshey RM (2003). Bacterial motility on a surface: many ways to a common goal. Annu Rev Microbiol.

[B20] Wood TK, Gonzalez Barrios AF, Herzberg M, Lee J (2006). Motility influences biofilm architecture in *Escherichia coli*. Appl Microbiol Biotechnol.

[B21] Fontaine F, Stewart EJ, Lindner AB, Taddei F (2008). Mutations in two global regulators lower individual mortality in *Escherichia coli*. Mol Microbiol.

[B22] Lane MC, Lockatell V, Monterosso G, Lamphier D, Weinert J, Hebel JR, Johnson DE, Mobley HLT (2005). Role of motility in the colonization of uropathogenic *Escherichia coli *in the urinary tract. Infect Immun.

[B23] Ciche TA, Kim K, Kaufmann-Daszczuk B, Nguyen KCQ, Hall DH (2008). Cell invasion and matricide during *Photorhabdus luminescens *transmission by *Heterorhabditis bacteriophora *nematodes. Appl Environ Microbiol.

[B24] Xu J, Hurlbert RE (1990). Toxicity of irradiated media for *Xenorhabdus *spp. Appl Environ Microbiol.

[B25] Brachmann AO, Joyce SA, Jenke-Kodoma H, Schwar G, Clarke DJ, Bode HB (2007). A type II polyketide synthase is responsible for anthraquinone biosynthesis in *Photorhabdus luminescens*. Chembiochem.

[B26] Philippe N, Alcaraz J-P, Coursange E, Geiselmann J, Schneider D (2004). Improvement of pCVD442, a suicide plasmid for gene allele exchange in bacteria. Plasmid.

